# Demand Forecasting of Nurse Talents in China Based on the Gray GM (1,1) Model: Model Development Study

**DOI:** 10.2196/59484

**Published:** 2024-08-14

**Authors:** XiuLi Wu, Aimei Kang

**Affiliations:** 1 Hubei Province Key Laboratory of Occupational Hazard Identification and Control Institute of Nursing Research, School of Medicine Wuhan University of Science and Technology Wuhan Hubei China; 2 Department of Nursing Wuhan Asia General Hospital Affiliated to Wuhan University of Science and Technology WuHan China

**Keywords:** nursing human resource, nursing manpower, Gray GM (1,1) model, forecasting, nursing

## Abstract

**Background:**

In a global context, the shortage of nursing personnel has emerged as a significant challenge, particularly in countries such as China experiencing population aging. The inadequacy of nursing human resources has become one of the primary threats affecting the quality of health services available to Chinese residents. Therefore, forecasting the demand for nursing personnel has become an important issue.

**Objective:**

This study presents a Gray GM (1,1) forecasting model for predicting the future 10-year demand for nursing workforce and the number of specialized geriatric nurses, aiming to provide a scientific basis for the development of policies in health care institutions in China.

**Methods:**

Based on data from the China Statistical Yearbook 2022, the Gray GM (1,1) model was used to predict the demand for nursing jobs and geriatric nurses over the next 10 years (2024-2033).

**Results:**

The results indicate that from 2024 to 2033, amidst a continuous growth in the overall population and an increasingly pronounced trend of population aging, the demand for nursing workforce in China, especially for specialized geriatric nurses, is projected to steadily increase.

**Conclusions:**

The paper provides a reference basis for the establishment of China’s health care workforce system and the involvement of government departments in health care workforce planning.

## Introduction

Health human resources are an essential foundation for achieving universal health coverage and the United Nations Sustainable Development Goals [1]. In 2016, the World Health Organization (WHO) released the “Global Strategy on Human Resources for Health: Workforce 2030” [2] with a vision to strengthen health systems to ensure equitable access to health workforce services and accelerate progress toward universal health coverage and the UN Sustainable Development Goals. Following this, in 2022, the “National Nursing Career Development Plan (2021-2025)” [3] issued by China’s National Health Commission explicitly expressed the crucial role of nursing human resource planning in achieving universal health coverage and sustainable development goals. Therefore, the comprehensive promotion of high-quality nursing development and the improvement of the population’s health level are among the primary development goals of China's future medical system. In China, registered nurses [4] are individuals who have completed relevant nursing professional education, obtained a nursing qualification certificate, and are registered and legally licensed to practice in medical institutions, including hospitals, health centers, community health service centers, clinics, and nursing homes. As direct providers of medical nursing services in China, registered nurses have always been an indispensable part of Chinese medical institutions, playing a key role in ensuring patient health and medical safety, and contributing significantly to advancing the construction of health development goals worldwide [5]. Despite increasing attention and implementation of measures such as improving remuneration and optimizing career advancement pathways to prevent the loss of nursing human resources in recent years, the issue of nursing staff shortage and uneven distribution in China has remained prominent [5].

Registered nurse density per 1000 population [5] refers to the number of registered nurses per 1000 permanent residents, indicating the availability and capacity of health care services within a particular country or region [6]. A higher value implies better assurance of public health needs in that area. The “2020 World Nursing Report” [7] highlights significant disparities in nurse supply among countries. According to WHO statistics, Sweden has the highest number of nurses per capita globally, with 21.67 nurses per thousand people [8]. Norway follows closely with 18.35 nurses per thousand people or more, while the United States and Japan have 15.69 and 12.7 nurses per thousand people, respectively [8]. Conversely, China’s registered nurse density stands at only 3.56 nurses per 1000 population, revealing a notable gap compared with the developed nations [9]. In addition, the increasing trend of aging population in China has indeed brought about a growing pressure on the demand for health care services [10]. In 2021, the population aged 65 years and older in China accounted for 14.2% of the total population, indicating a doubling of the proportion of older individuals from 7% to 14% over 21 years. It is projected that by 2050, the population aged 60 years and older in China will increase to 478.9 million. With the increasing proportion of the older adult population in China, the demand for caregiving services is correspondingly rising [11].

In previous studies, it has been widely reported that an increased presence of registered nurses significantly improves patient outcomes and plays a crucial role in ensuring patient safety [12]. However, in recent years, some scholars have raised concerns regarding this viewpoint. They argue that an excessive number of nurses could lead to additional expenses for health care institutions and wastage of manpower [13]. Some studies even indicate a correlation between an excessive allocation of nursing staff and poorer quality of care [14]. Such as Park’s Optimized Nurse Staffing (Sweet Spot) Estimation Theory emphasizes the crucial balance between nursing quality, cost, and staffing levels, making a significant contribution to enhancing the effectiveness of health care workforce planning [15]. As widely known, effective health care workforce planning drives the establishment of resilient and sustainable health care systems [16], with workforce demand forecasting playing a crucial role in health care workforce planning [17]. Therefore, to clarify the current status of nursing human resource allocation in China, further seeking the balance point between nursing quality, cost, and the level of nurse staffing, it is essential to conduct accurate forecasting and analysis of the future demand for nurses in China. It is worth noting that Park et al [18] suggested integrating mathematical programming into nursing research to assist nursing leaders and managers in determining optimal nurse staffing and composition. Consequently, adopting mathematical models to predict the demand for nursing personnel is highly feasible.

Currently, many scholars in health care research use mathematical models and algorithms for predictive purposes. The methods are mainly divided into 3 categories: first, machine learning models for demand prediction, such as the study by Vollmer research team in 2021, which developed a machine learning–based model to predict emergency department demand [19]; in 2022, Soltani et al [20] used deep machine learning models to predict the demand of patients with end-stage cancer at home. Second, time series models, such as using time series analysis to investigate depression rates during the COVID-19 crisis in Peru [21]; Zhang et al [5] used time series analysis to assess the impact of the “National Nursing Development Plan” on the nursing human resources in China, concluding that the implementation of the plan significantly expanded the scale of nursing human resources and optimized allocation efficiency. Third, the use of hybrid models, such as Chung development of a dynamic model to forecast the demand for cancer nurses over the next decade [17]. Human resources for nurses are influenced by various external and internal factors, such as economic conditions, industry development trends, and environmental changes, as well as organizational strategic goals, business requirements, and talent adjustment policies [22]. Therefore, nurse workforce forecasting requires flexibility and adaptability, aiming for rapid and accurate predictions through simple means whenever possible. The aforementioned machine learning models, time-series models, and hybrid models have demonstrated good predictive performance. However, they generally require large amounts of historical data and involve complex processes. Although data accumulation on nursing human resources in China has been relatively extensive to date, nurse workforce dynamics are influenced by various factors such as national policies and socioeconomic conditions. Consequently, historical data accumulated over the long term may not be applicable to the current situation. Therefore, there is a need to identify a model that does not rely on extensive data. Fortunately, the Gray GM (1,1) model [23] provides a structurally simple and widely applicable mathematical forecasting model, using a small amount of data to forecast within an unknown required data range. Due to its high predictive accuracy, good performance, and convenience, the Gray GM (1,1) model has been widely applied in fields such as construction, ecological environment, and the medical industry [23], especially during the COVID-19 crisis, with many scholars applying it to COVID-19 prediction and achieving outstanding results [24]. In terms of human resource forecasting, relevant studies can be traced back to 2007 when Lin et al [25] used the gray model to forecast the long-term demand and supply of nursing staff in Taiwan, showing good results. However, as the data included in this study are limited to the Taiwan region, it is not applicable to predict the nursing workforce in mainland China. Therefore, building upon previous research, this study uses the latest data to construct a Gray GM (1,1) model for forecasting the demand for nursing positions in mainland China over the next 10 years. Furthermore, incorporating current social development trends, an analysis of the forecast results is provided.

## Methods

### Data Source

The research object was registered nursing talent resources per thousand people in China. Data were extracted from the China Statistical Yearbook 2022 [26] from which the total population and registered nurse numbers were collected from 2008 to 2021, and the registered nurses per thousand population were calculated.

### Procedures

First, we used Excel 2019 to enter and process the raw data and used descriptive statistical methods to study the dynamic trends of registered nursing talent resources per thousand people in China. Subsequently, the Gray GM (1,1) model was applied to model the registered nursing talent resources per thousand population, and the model was used to forecast the demand of these resources over the next 10 years. In addition, we have also forecasted the demand for geriatric nurses in China. Finally, we used the MATLAB 2023 software (MathWorks Inc) to design a model program for fitting and prediction, and the accuracy of the predictions was evaluated.

### Ethical Considerations

All procedures in this study are conducted in accordance with the guidelines of our institutional ethics committee and the principles of the Declaration of Helsinki. Since all data are extracted from public databases, informed consent is not required for the use of these data. All research results will be reported accurately and truthfully, and the confidentiality and security of the data will be ensured. This study has been approved by the Ethics Review Committee of the School of Medicine, Wuhan University of Science and Technology (ID 2024097), for both the study and the entire research protocol.

## Results

### The Development of Nurse Practitioners in China From 2008 to 2021

According to the data from the “China Statistical Yearbook 2022 [26],” as shown in Table 1, the total number of registered nurses in China has been increasing annually from 1,678,091 in 2008 to 5,019,422 in 2021, with an average annual growth rate of 8.14%. The number of registered nurses per thousand people in the country increased by 2.29, with an average annual growth rate of 7.64%.

**Table 1:** Development of the number of practicing nurses per 1000 national population from 2008 to 2021.

Year	Total population (unit: 10,000 persons)	Number of registered nurses (unit: person)	Number of nurses per thousand population (unit: person)
2008	1,32,802	16,78,091	1.27
2009	1,33,450	18,54,818	1.39
2010	1,34,091	20,48,071	1.53
2011	1,34,916	22,44,020	1.66
2012	1,35,922	24,96,599	1.85
2013	1,36,726	27,83,121	2.04
2014	1,37,646	30,04,144	2.20
2015	1,38,326	32,41,469	2.37
2016	1,39,232	35,07,166	2.54
2017	1,40,011	38,04,021	2.74
2018	1,40,541	40,98,630	2.94
2019	1,41,008	44,45,047	3.18
2020	1,41,212	47,08,717	3.34
2021	1,41,260	50,19,422	3.56

### Population Forecast in China

Construction of Chinese population Gray GM (1,1), the prediction model. Take the population number from 2008 to 2021 as the original sequence:



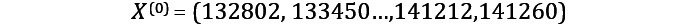

**(1)**


**Step 1:** First accumulation of raw data *X*^(0)^:



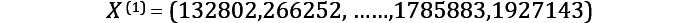

**(2)**


**Step 2:** Neighborhood mean generation was performed on the sequences after cumulative generation. Neighborhood mean generation is an equivalence time series, and new data are generated with the average construction of adjacent data. Set the newly generated neighbor mean sequence *Z*, yielding:



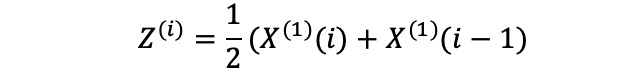

**(3)**


**Step 3:** Construct data matrix *B* and data vector *Y*:



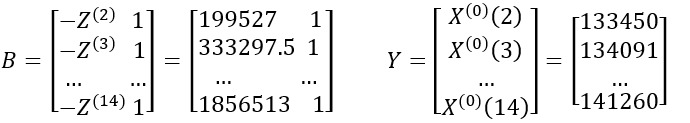

**(4)**


**Step 4:** Calculate the development coefficient â and the Gray System residual 
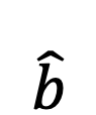
:



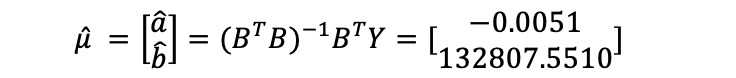

**(5)**


Calculation results indicate that: â=–0.0051 
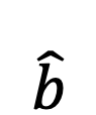
=132807.551

**Step 5:** Establish a model to solve the time-response function and predict it.

The whitening equation under the Gray GM (1,1) model “*X*^(0)^(*k*) *+aZ*^(1)^*k=b* ”is:



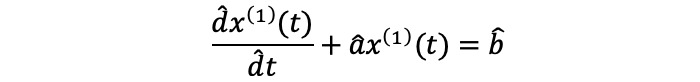

**(6)**


**Step 6:** Generate the model:



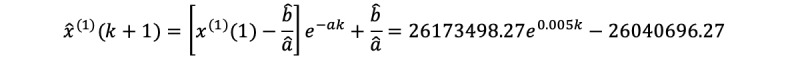

**(7)**


**Step 7:** Find the generated sequence prediction value *
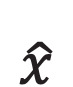
*^(1)^(*k+1*) and model reduction values *
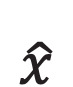
*^(0)^(*k*+1)：Bring *k*=0,1, 2..., 14 into the model to calculate *X*^(1)^, among them take *
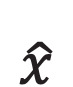
*^(0)^(1) *= 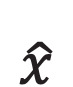
* ^(1)^(1)=132802, got by the formula : *
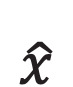
*^(0)^(*k*+1)= *
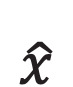
*^(1)^(*k*+1)-*X*^(1)^*k*:



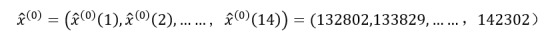

**(8)**


Calculate the simulated values.

**Step 8:** Evaluation of the model-fitting effect.

Using MATLAB 2023 software, the fitting results were tested using the posterior difference test method. In the gray prediction model of the national total population, the posterior difference ratio C value was 0.0226, and the small probability error *P* value was equal to 1.000. Table 2 shows that the model has high accuracy and a good fitting effect.

**Table 2:** Evaluation table of posterior difference ratio and probability of small error.

Predicting rank accuracy	*P* value	*C* value
Good	≥.95	≤.35
Qualified	.8, .95	.35, .5
Manage with an effort	.7, .8	.5, .65
Unqualified	≤.7	>.65

**Step 9:** Model prediction

The established model can predict the total population value for the next decade (2024-2033).

### Population-to-Nurse Ratio Prediction

To predict the number of registered nurses per thousand population, Gray GM (1,1), model, input model code, and annual data were input into the MATLAB software, and the model was established as follows:



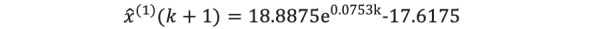

**(9)**


The posterior difference ratio *C* value was 0.005, and the small error probability *P* value was 1.000, with a better model accuracy level.

### Forecast Results of Nurse Demand Under the Total Population

The above prediction results for the future population size population-nurse ratio were added into the formula: demand for registered nurses in a certain year = population forecast (10,000) 10 predicted number of nurses per 1000 population to obtain the number of demands for registered nurse resources in the next 10 years (Table 3). As can be seen from Table 3, the Gray GM (1,1) model predicts that by 2032, the total population will exceed 1.5 billion, the population of nurses per 1000 people will reach 8.357, and the demand for registered nurses will rise to 12.58 million.

**Table 3:** Forecast results of total population and demand number of nurses between 2024 and 2033.

Year	Population measurement (unit：10,000 person)	Number of nurses per thousand population (unit：person)	Nurse requirements (unit：person)
2024	144503.493	4.574	66,09,590
2025	145244.674	4.932	71,63,467
2026	145989.656	5.318	77,63,730
2027	146738.460	5.734	84,13,983
2028	147491.104	6.183	91,19,375
2029	148247.609	6.666	98,82,185
2030	149007.993	7.188	107,10,695
2031	149772.279	7.751	116,08,849
2032	150540.484	8.357	125,80,668
2033	151312.629	9.011	136,34,781

### Demand Forecast Results of Nurses Under the Trend of Population Aging

Our study investigates the aging population trend in China in recent years, which has shown a significant shift toward an older demographic structure. Population aging is recognized as a crucial factor that influences the demand for nursing professionals in our country [10]. In light of this, we used the Gray GM (1,1) model and analyzed data from the China Statistical Yearbook 2022 to predict the future numbers of the older adult population and the corresponding demand for nurses in the age group from 2024 to 2033 in China. The detailed forecast results are shown in Table 4. In 2033, the older adult population in China can reach 350.39 million, the number of nurses per 1000 older adult population is nearly 9, and the requirement of geriatric nurses reached 31,33,882. Owing to the slow growth of the older adult population from 2008 to 2018, the predicted total older adult population is likely to be underestimated.

**Table 4:** Forecast results of total older adult population and demand of nurses from 2024 to 2033.

Year	Total older adult population (unit：10,000 person)	Number of nurses per thousand older adult population (unit：person)	Requirements of geriatric nurses (unit：person)
2024	22704.306	4.544	10,31,684
2025	23825.734	4.899	11,67,223
2026	25002.553	5.282	13,20,635
2027	26237.498	5.695	14,94,226
2028	27533.441	6.14	16,90,553
2029	28893.394	6.619	19,12,454
2030	30320.519	7.137	21,63,975
2031	31818.133	7.695	24,48,405
2032	33389.719	8.296	27,70,011
2033	35038.929	8.944	31,33,882

### Visual Analysis of the Demand Proportion of Geriatric Nurses

**Figure 1:**
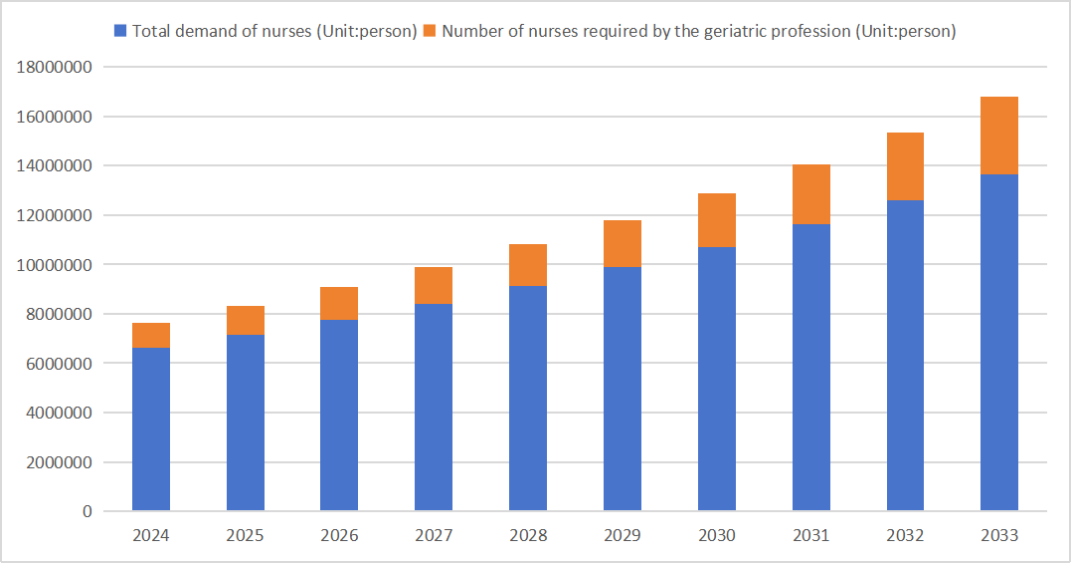
The proportion of geriatric nurse demand from 2024 to 2033.

By using the prediction data generated through the gray model constructed in the paper, we can conduct a comparative analysis between the demand for geriatric nurses and the total demand for nurses in the next decade, as shown in Figure 1. From Figure 1, it can be observed that the demand for geriatric nurses in China is projected to steadily rise over the next 10 years. By the year 2024, the demand is estimated to be more than 1.03 million, and by 2033, it is anticipated to reach more than 3.13 million. This growth trend is primarily attributed to the aging population in China and the increasing demand for older adult care services. Furthermore, a more in-depth analysis reveals that the proportion of geriatric nurses within the overall demand for nurses is increasing annually from 2024 to 2033. In 2024, geriatric nurses constitute around 10% of the total, while this proportion is expected to rise to 20% by 2033. This indicates that in the coming decade, the role of specialized geriatric nurses in China’s nursing workforce will become increasingly significant. In conclusion, the demand for geriatric nurses in China is projected to steadily increase over the next decade, with their proportion within the nursing workforce rising annually. To address this trend, it is imperative for China to enhance the training and education of geriatric nurses to meet the growing demand for older adult care services. Simultaneously, attention must also be given to the overall expansion of the nursing workforce to ensure the quality and coverage of health care services.

## Discussion

### Principal Findings

The research findings indicate that from 2008 to 2021, the total number of registered nurses in China experienced a rapid overall growth rate, with an average annual growth rate of 8.14%. The number of registered nurses per thousand population increased from 1.27 to 3.56. Based on the forecast results from Tables 3 and 4, under the gradual increase in the nurse-to-population ratio, by 2033, the number of registered nurses per thousand population in China is projected to reach 9.01, and the number per thousand older adult population could reach 8.94, approaching the current number in the United States. However, it should be noted that the growth in nurse numbers is also influenced by certain external factors not accounted for in this model, potentially introducing bias into the forecast results.

### The Total Workforce of Nurses in China Is Steadily Increasing, Yet There Remains a Shortage Compared With the Demand for Medical Services

In recent years, with the support of the government and collaboration among health departments, the nursing industry in China has experienced rapid development and achieved significant outcomes. By the end of 2021, the proportion of registered nurses in China with a graduate degree had increased to 0.3% (14,900/5,019,422) [5]. The proportion holding bachelor’s degrees has risen to 34.2% (17,16,645/50,19,422), and the proportion of those with university education has reached 47.2% (23,69,168/50,19,422) [5]. However, despite the improvement in the quality of nursing talents, the scarcity of nursing professionals in China persists. Currently, the total number of registered nurses in China exceeds 5.2 million, but the ratio of registered nurses per thousand people is only about 3.7, below the WHO’s recommended standard of 5 nurses per thousand people. According to the “Healthy China 2030” planning outline [27], it is projected that by 2030, China will need 4.7 registered nurses per thousand people, indicating a need for nearly 2 million more nurses to fill this gap. Furthermore, factors such as the aging population in developing countries and the rapid increase in the number of patients with chronic disease will not only increase the demand for nursing care but also worsen the severe shortage of nursing professionals [28]. There is a significant gap in the number of nurses in China; therefore, it is necessary to formulate relevant guiding policies and coordinate the planning of the development of the nursing profession in China to meet the growing demand for clinical nursing services among the population.

### The Population Aging Trend Increases the Demand for the Geriatric Medical Care Service Model

As China’s economy and society continue to develop, population aging has become an inevitable trend [10]. According to this study’s predictions, by 2033, China’s older adult population will reach 360 million, with a demand for 3 million nurses in the older adult care sector. Compared with younger individuals, the nursing needs of older adults are diverse and multilayered, requiring higher comprehensive qualities from geriatric nurses [29]. Therefore, nurturing geriatric nursing personnel has become a focal point in the development of the nursing profession. However, despite the severity of the aging society, there still exists a lack of relevant training in geriatric nursing within the nursing profession. For instance, a study by Nawagi et al [30] revealed that almost no courses on geriatric nursing knowledge or improving care capabilities for older patients were incorporated into nurses’ training at all levels. This rings alarm bells for China to advance the development of geriatric nursing. Strictly speaking, we should focus on addressing the deficiencies in the nursing field and promote the comprehensive development of geriatric nursing to meet the needs of an aging society. In addition, it is suggested to optimize the existing medical nursing service model and vigorously develop long-term care and home care service models that can meet the nursing needs of older adults, promoting collaboration among disciplines such as nursing, rehabilitation, medicine, and social work, and continuously enhance the skill levels of clinical caregivers to meet the diverse care needs of the older adult population [31].

### Exploring New Approaches Actively to Promote the Development of Nursing Education in China

Undoubtedly, there is a strong link between education and human resources [32]. High-quality education provides a solid foundation for the development of nursing human resources. In recent years, numerous studies have highlighted the role of nursing education in addressing nursing shortages, and governments worldwide have actively implemented various measures to develop nursing education in response to the shortage of nurses in their countries [22]. The development of computer and software technology offers infinite possibilities for nursing practice, and we must adapt to and embrace teaching methods that are relevant to the new generation [33]. The roles of virtual reality technology and artificial intelligence in fostering nursing students’ cognition and skill mastery have been further confirmed, and their development in the education sector is receiving increasing attention [34,35]. However, for many researchers and educators, they still remain quite novel and unfamiliar [35]. Therefore, we recommend that health institutions and education departments increase investment and support for new technologies such as artificial intelligence in nursing education, comprehensively elevate the level of nursing education and practice in China, and better meet society’s demand for nursing talent [36]. Second, moreover, according to a study in China [37], only a small percentage of high school students are willing to pursue nursing education. This low professional recognition undoubtedly exacerbates the shortage of nursing professionals. The research also indicates that in China, nurses’ roles often depend heavily on doctors, inevitably leaving the public with stereotypical impressions such as “mechanical” and “hectic,” which significantly diminishes the professional identity of nursing [37]. Therefore, it is imperative to promote the reshaping of the nursing profession’s image, enhance public awareness regarding the societal stature of nurses, and attract more talents to the field of nursing.

### Statistical Prediction Provides Strong Evidence for Optimizing the Resource Allocation in the Health Industry

Statistical forecasting [38] applies statistical principles and methods to predict and analyze future trends and developments within specific domains. By collecting data and using appropriate statistical models, forecasters can predict future trends and provide scientific evidence for decision-making. Its characteristics include objectivity, scientific rigor, and forward-looking perspective [23]. This study uses the gray model to forecast the demand for nursing personnel in China, estimating the overall trend and quantity of future nursing personnel based on limited historical data. Compared with other forecasting methods, the gray model requires less data, thus offering broad application prospects in manpower forecasting. However, gray models are relatively adept at handling linear or approximately linear relationships. Considering this, we suggest that in practical applications, adjustments and corrections should be made to the predictive results based on real-world circumstances. In the future, this model can be further optimized in terms of data processing and parameter optimization to enhance overall accuracy [23].

### Conclusions

The study proposes a gray forecast model to predict the nursing manpower demand in China over the next 10 years, with a specific focus on the projected quantity of geriatric nurses amidst the trend of population aging. Our findings underscore the immediate need for health care and educational organizations to implement improvement measures to bridge the gap between the supply and demand for nurses. In addition, we have identified the future required number of nurses, providing a reference basis for governmental involvement in health care workforce planning.

### Limitations

Similar to many studies, this research also has its limitations. The study exclusively uses a gray model to forecast the overall nursing demand in China, without considering the impact of geographical disparities on nursing resource allocation. Given China’s vast geographical expanse, there are significant differences in nursing resource distribution among regions. Developed areas may have higher levels of nurse manpower resources than impoverished regions, which could lead to inaccuracies in the forecasting results due to regional imbalances. In addition, factors, such as low birth rates, nurse migration, and the increase in patients with chronic disease, also significantly influence nursing demand. Therefore, future research will explore the impact of these factors on nursing manpower and continue to refine relevant models for precise forecasting.

## Abbreviations

WHO: World Health Organization

## Data Availability

The main data source of this study is China Statistical Yearbook, which has been cited in the text. As an authoritative compilation of statistical data, the report can also serve as an important reference source of information for other studies.
